# Thioredoxin-Interacting Protein’s Role in NLRP3 Activation and Osteoarthritis Pathogenesis by Pyroptosis Pathway: In Vivo Study

**DOI:** 10.3390/metabo14090488

**Published:** 2024-09-07

**Authors:** Ruba Altahla, Xu Tao

**Affiliations:** Department of Rehabilitation, Tongji Hospital, Tongji Medical College, Huazhong University of Science and Technology, Wuhan 430030, China; rubamntahla91@gmail.com

**Keywords:** TXNIP, NLRP3, osteoarthritis, pyroptosis pathway

## Abstract

Thioredoxin-interacting protein (TXNIP) has been involved in oxidative stress and activation of the NOD-like receptor protein-3 (NLRP3) inflammasome, directly linking it to the pyroptosis pathway. Furthermore, pyroptosis may contribute to the inflammatory process in osteoarthritis (OA). The purpose of this study was to investigate the role of TXNIP in activating the NLRP3 inflammasome through the pyroptosis pathway in an OA rat model. Destabilization of the medial meniscus (DMM) was induced in the OA model with intra-articular injections of adeno-associated virus (AAV) overexpressing (OE) or knocking down (KD) TXNIP. A total of 48 healthy rats were randomly divided into six groups (N = 8 each). During the experiment, the rats’ weights, mechanical pain thresholds, and thermal pain thresholds were measured weekly. Morphology staining, micro-CT, 3D imaging, and immunofluorescence (IF) staining were used to measure the expression level of TXNIP, and ELISA techniques were employed. OE-TXNIP-AAV in DMM rats aggravated cartilage destruction and subchondral bone loss, whereas KD-TXNIP slowed the progression of OA. The histological results showed that DMM modeling and OE-TXNIP-AAV intra-articular injection caused joint structure destruction, decreased anabolic protein expression, and increased catabolic protein expression and pyroptosis markers. Conversely, KD-TXNIP-AAV slowed joint degeneration. OE-TXNIP-AVV worsened OA by accelerating joint degeneration and damage, while KD-TXNIP-AAV treatment had a protective effect.

## 1. Introduction

Osteoarthritis (OA) pathogenesis involves various factors, including inflammation, autophagy, senescence, programmed cell death, and metabolic disorders. Among these, programmed cell death and inflammatory processes play crucial roles in regulating the onset and progression of OA [1,2]. Pyroptosis, a form of inflammatory programmed cell death caused by the activation of caspase-1, has been identified as a significant pathogenic mechanism in OA. The activation of the NOD-like receptor family pyrin domain containing 3 (NLRP3) inflammasome and the subsequent induction of pyroptosis contribute to cartilage degradation, synovial inflammation, and the overall progression of OA [3]. Mechanical stress, oxidative stress, and inflammatory mediators can trigger pyroptosis in joint tissues, thereby perpetuating the inflammatory cycle in OA. In OA, the activation of the pyroptosis pathway through inflammasome activation results in the release of pro-inflammatory cytokines such as interleukin-1 beta (IL-1β) and interleukin-18 (IL-18), which contribute to cartilage degradation, synovial inflammation, and overall joint damage [4,5].

Mitigating these pathogenic factors could potentially restore chondrocyte metabolic function and aid in improving OA. However, effective treatment methods targeting these underlying pathogenic mechanisms still require further elucidation.

Thioredoxin-interacting protein (TXNIP) is a multifunctional protein that plays a significant role in maintaining cell integrity through its involvement in various cellular processes, including proliferation, differentiation, autophagy, pyroptosis (a form of programmed cell death), and inflammation. TXNIP also modulates gene expression, metabolism, and redox reactions [6,7]. While TXNIP is often upregulated in diseases such as diabetes [8], cardiovascular disorders [9], and neurodegenerative conditions [10,11], it tends to be downregulated in certain types of cancer [12]. TXNIP plays a substantial role as a metabolic regulator, particularly in lipid and glucose metabolism.

Of particular relevance to OA is the connection between TXNIP and the pyroptosis pathway. TXNIP plays a definitive role in activating the NLRP3 inflammasome, a key regulator of the pyroptosis pathway [13]. Thus, TXNIP triggers the cleavage and activation of caspase-1. Once activated, caspase-1 plays a dual role in the pyroptosis pathway. First, it cleaves the precursor forms of IL-1β and IL-18, converting them into their mature forms. Second, caspase-1 directly induces pyroptotic cell death by disrupting the cell membrane and releasing cellular contents [14]. In the context of OA, chronic oxidative stress and inflammatory processes contribute to articular cartilage degeneration. Previous studies evaluating the effects of medicinal plants on TXNIP downregulation in OA tissues have demonstrated that these plants can hinder OA progression by inhibiting the endoplasmic reticulum stress (ERS)-inositol-requiring enzyme 1α (IRE1α)–TXNIP–NLRP3 axis [15]. Furthermore, the TXNIP–NLRP3–caspase-1 axis has been implicated in the production of pro-inflammatory cytokines, such as IL-1β, that can exacerbate cartilage damage and joint inflammation in OA. Overall, the biological function and precise role of TXNIP in the pathogenesis of OA remain active areas of research, with the dysregulation of the pyroptosis pathway emerging as a key mechanism of interest. Accordingly, our study aims to investigate the role of TXNIP in activating the NLRP3 inflammasome through the pyroptosis pathway in OA using a DMM rat model.

## 2. Materials and Methods

### 2.1. Animals

Forty-eight Sprague-Dawley (SD) male rats averaging 6 weeks old (220 ± 10 g) were used. The Experimental Animal Centre of Tongji Medical carried out the administration of this training protocol. The experimental animals were kept in a special pathogen-free (SPF) environment to maintain a high standard of hygiene for the duration of the long-term experiments. This study received ethical approval from the Ethics Committee under the authorization number TJH-202210037. Animal pairs were kept in plastic cages furnished with sterilized sawdust bedding in a controlled environment preserved at a constant temperature of 22 ± 2 °C and a humidity level of 60 ± 5%. The animals experienced a 12-h light–dark cycle, alternating between 12 h of light and dark each. The rats had unlimited access to food and water with unrestricted mobility within their housing. All the SD rats and experimental procedures strictly observed the appropriate SD rat protection and usage regulations, established by the International Association for the Study of Pain. An ARRIVE checklist has been integrated to exhibit adherence to the ARRIVE guidelines.

### 2.2. Experimental Materials

The TXNIP-overexpressing plasmid and TXNIP overexpression and knockdown adeno-associated virus (AAV) vectors were generated by GeneChem Co. Ltd. in Shanghai, China (overexpressed adeno-associated virus (TXNIP), GOSV0395090); (RNAi-custom-adeno-associated virus (TXNIP), GIDV0395091) (Supplementary Materials).

### 2.3. OA Model and AVV Interarticular Injection

The six groups included 48 male SD rats. The SHAM group (N = 8) underwent sham operation (control group), while the 40 rats used to induce OA underwent destabilization of the medial meniscus (DMM) surgery on their right hind limbs. The DMM group (N = 8) did not receive additional AAV injections. Two weeks after surgery, the 32 DMM rats were randomly assigned to receive intra-articular injections (20 µL) of one of the following treatments: overexpression negative control AAV (OE-NC, N = 8); overexpression TXNIP AAV (OE-TXNIP, N = 8); knockdown negative control AAV (KD-NC, N = 8); or knockdown TXNIP AAV (KD-TXNIP, N = 8). Four weeks after AAV injection, the rats were euthanized for further experimentation. Throughout the study, behavioral assessments including body weights, mechanical pain thresholds, and thermal pain thresholds were recorded weekly for all of the rats. Furthermore, micro-CT scans, histological staining, and immunohistochemistry (IHC) were conducted; immunofluorescence (IF) staining was conducted on joint sections to evaluate the TXNIP expression level in SHAM/DMM rat articular cartilage among experimental groups; and enzyme-linked immunosorbent assay (ELISA) was performed on rat sera.

### 2.4. Micro-Computed Tomography and 3D

Following euthanasia, the entire right knee joints were fully excised and subjected to scanning (a micro-CT 50 Scanco Medical, Bassersdorf, Switzerland). The scanning criteria were as follows: a voxel size of 10.5 µm, 100 kV voltage, and 98 µA current. The micro-CT system’s integrated evaluation system was utilized to obtain CT, 3D, and corresponding data, including bone volume/tissue volume (BV/TV), trabecular thickness (Tb.Th), trabecular number (Tb.N), and trabecular separation (Tb.Sp).

### 2.5. Histological Analysis

A decalcification process was performed after the micro-CT using 10% ethylenediaminetetraacetic acid (EDTA). After dehydration, using a series of increasing ethanol concentrations, the sample was embedded in paraffin. Sections were obtained from the joint in the sagittal plane, with a section thickness of 4 µm. These slices were gathered for histological analysis. The articular cartilage tissue samples were examined under a microscope to assess their morphological characteristics. The slices were dyed with hematoxylin eosin and safranin O/fast green, following the producer’s instructions. The extent of cartilage injury at the histopathological degree in SHAM/DMM (SD) rats was evaluated by the scoring system of the OA Research Society International (OARSI) [16]. This scoring system, ranging from 0 to 6 points, is recognized for its remarkable sensitivity and reliability in evaluating the histopathological features of cartilage in DMM rat models.

### 2.6. Immunohistochemistry (IHC)

The joint sections were formed in the sagittal plane, and the thickness of each slice was 4 µm, specifically for immunohistochemical analysis. Tissue expression assessment was conducted using the IHC technique in Matrix MetalloPeptidase 13 (MMP13, rabbit, 1:800; A-A14); collagen type II (Col2a1, rabbit, 1:800; A-A04); cysteinyl aspartate specific proteinase 1 (caspase-1, rabbit, 1:200; ER1905-47); gasdermin D (GSDMD, rabbit, 1:200; ER1901-37); and NOD-like receptor protein-3 (NLRP3, rabbit, 1:200, ET1610-93). Moreover, the digital image analysis technique was used—specifically, the (Image-Pro Plus-8) software—to examine the cell percentage demonstrating positive staining in the cartilage sections.

### 2.7. Immunofluorescence (IF)

The tissue samples were processed as follows: The tissues were placed in paraffin and mounted onto slides, following the established protocol. The prepared slides were sustained in incubation with the primary antibodies (TXNIP, rabbit, 1:200, ET1705-72). Following the primary antibody incubation, the slides were treated for one hour with the corresponding secondary antibodies (from Boster). The slides were stained for 10 min with diamidino-2-phenylindole (DAPI, provided by Boster). Finally, the IF images were acquired by EVOS FL Auto Imaging. This systematic approach allowed for the visualization and analysis of the target proteins and cellular components within the tissue samples.

### 2.8. ELISA

Before euthanasia, isoflurane was used as an anesthesia option in the animals. After euthanasia, the blood supernatant was obtained through a cardiac puncture and temporarily preserved at a temperature of −80 °C for subsequent analysis. Serum levels of IL-18 (FY-EH-6593, Feiyue Biotechnology, Wuhan, China) and IL-1β (FY-EH6675, Feiyue Biotechnology, Wuhan, China) were quantified using ELISA assay kits following the instructions provided by the manufacturer.

### 2.9. Statistical Analyses

Statistical analyses for this study were conducted using GraphPad Prism 8 software, which generated statistical graphs. The data were expressed as mean ± SD, a one-way ANOVA was used for comparisons among multiple groups, and the post hoc Tukey test was used for multiple comparisons. The ordinal variables, such as the OARSI scores, were analyzed with the Kruskal–Wallis H test. Significance was set at *p* < 0.05.

## 3. Results

### 3.1. Behavior Assessment

**Figure 1:**
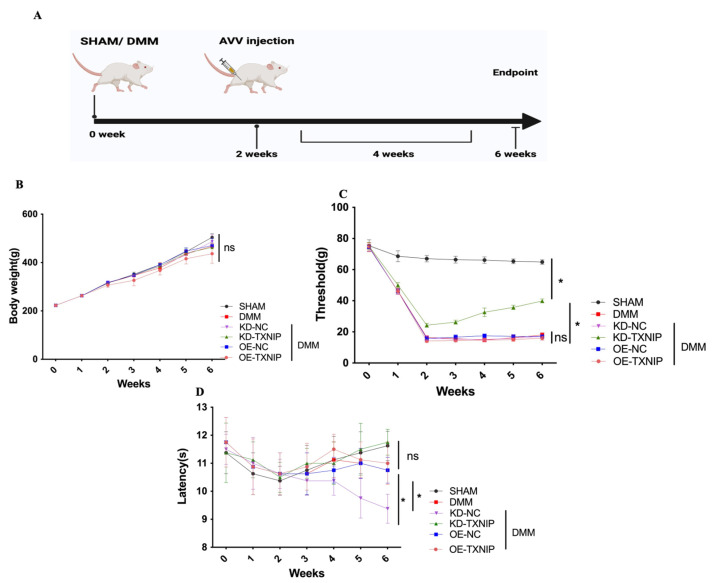
Behavior assessment. (**A**) The process diagram of SD rats receiving SHAM/DMM surgery and intra-articular AAV injection was created using Biorender.com; (**B**) the body weight of rats; (**C**) the mechanical pain assessment quantifications; (**D**) the thermal pain assessment quantifications. * *p* < 0.05.

The influence of TXNIP expression on OA was assessed using SHAM/DMM surgery in rats and intra-articular injection of AAV (Figure 1A).

Regarding body weight, there was no notable difference among the different groups (Figure 1B). Furthermore, after DMM surgery, the rats received KD-TXNIP treatment, which could effectively relieve mechanical pain symptoms (Figure 1C), and the thermal-pain assessment results did not demonstrate significant differences (Figure 1D).

### 3.2. Morphology Staining and Micro-CT, 3D

**Figure 2:**
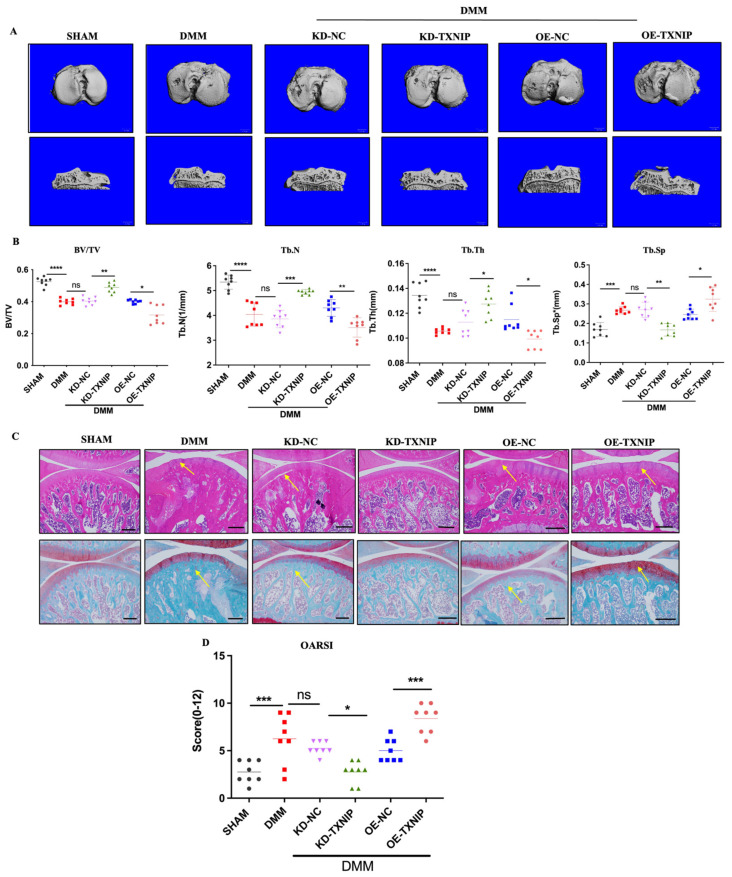
The CT-3D images of rats and histology staining. (**A**) The CT-3D images of rats after SHAM/DMM surgery and KD-NC, KD-TXNIP, OE-NC, and OE-TXNIP intra-articular injection; (**B**) the CT-3D quantification of the bone volume/tissue volume fraction (BV/TV), trabecular number (Tb.N), trabecular separation (Tb.Sp), and trabecular thickness (Tb.Th); (**C**) the hematoxylin and eosin; safranin O/fast staining quantification; (**D**) the OARSI score, white scalebar = 1 mm, Yellow arrow show the injury region, ns: non-significant, * *p* < 0.05, ** *p* < 0.01, *** *p* < 0.001, **** *p* < 0.0001, and N = 8.

To assess the effect of TXNIP on subchondral bone in the DMM group, CT-3D was conducted to examine alterations in the trabecular bone of the tibia (Figure 2A). OE-TXNIP showed increased cartilage and bone damage compared with the DMM surgery group. Conversely, KD-TXNIP mitigated cartilage degeneration, while the OE-TXNIP resulted in increased subchondral bone loss and accelerated degenerative alterations in microstructures after traumatic injury in the subchondral bone of the tibia, in contrast to the KD-TXNIP group. These observations were supported by the increased values of BV/TV, Tb.Th, and Tb.N and decreased values of Tb.Sp, as shown in (Figure 2B). Moreover, this trend matched the histological staining results (Figure 2C,D). In the DMM group, TXNIP-OE caused severe cartilage damage, chondrocyte disorder, and cartilage component loss rather than cartilage protection, which was demonstrated with KD-TXNIP.

### 3.3. TXNIP Upregulation Promotes Anabolic/Catabolic Disorders

**Figure 3:**
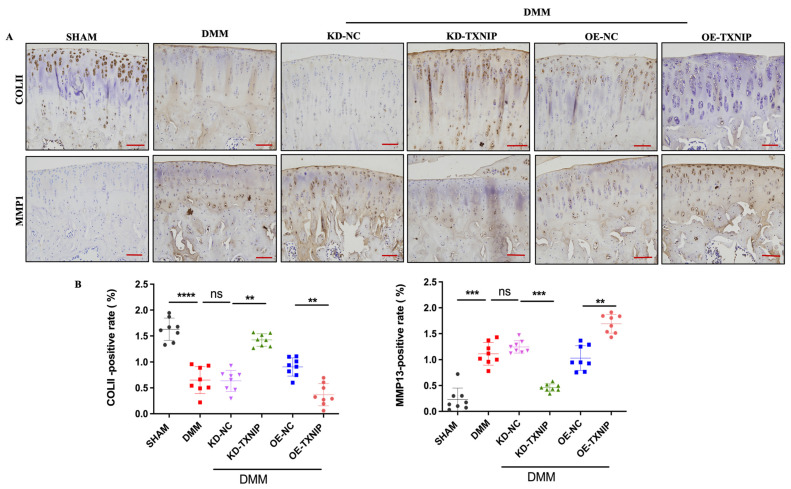
IHC images of rats. (**A**) Col II and MMP13 IHC images of rats after SHAM/DMM surgery and KD-NC, KD-TXNIP, OE-NC, and OE-TXNIP intra-articular injection of articular cartilage; (**B**) IHC quantification, (red scalebar = 200 μm), ns: non-significant, ** *p* < 0.01, *** *p* < 0.001, **** *p* < 0.0001, N = 8.

TXNIP-OE increased the MMP13-positive cells’ percentage and substantially decreased the col II-positive cells’ percentage compared with that of the DMM group, while the KD-TXNIP intra-articular injection restored chondrocyte anabolism (Figure 3A,B).

### 3.4. TXNIP Aggravates Chondrocyte Inflammation by Activating the Pyroptosis Pathway

**Figure 4:**
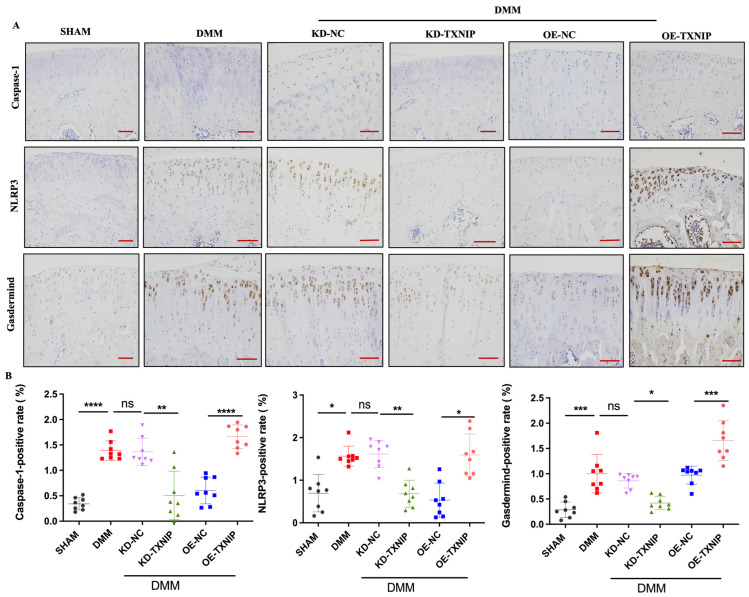
IHC images of rats. (**A**) Caspase-1, NLRP3, and gasdermin D IHC images of rats after SHAM/DMM surgery and KD-NC, KD-TXNIP, OE-NC, and OE-TXNIP intra-articular injection of articular cartilage; (**B**) IHC quantification (red scalebar = 200 μm), ns: non-significant, * *p* < 0.05, ** *p* < 0.01, *** *p* < 0.001, **** *p* < 0.0001, N = 8.

TXNIP-OE led to significantly increased caspase-1, NLRP3, and gasdermin D-positive chondrocytes compared with the levels of the DMM group, while KD-TXNIP intra-articular injection led to significantly decreased caspase-1, NLRP3, and gasdermin D-positive chondrocytes (Figure 4A,B).

### 3.5. TXNIP Regulation among SHAM/DMM Experimental Groups

**Figure 5:**
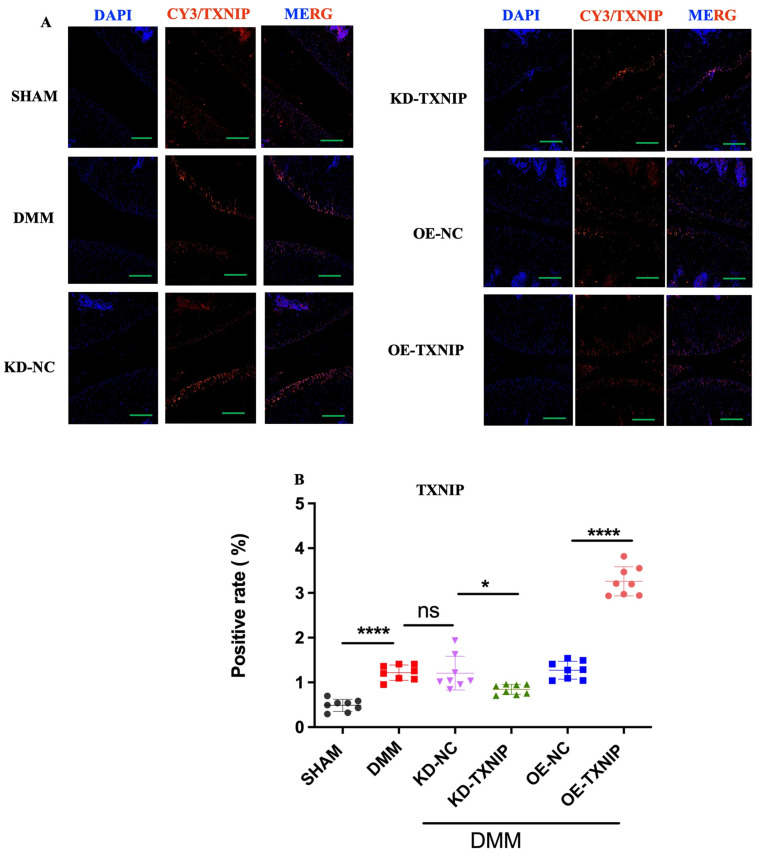
Immunofluorescence staining images of rats. (**A**) TXNIP IF images of rats after SHAM/DMM surgery and KD-NC, KD-TXNIP, OE-NC, and OE-TXNIP intra-articular injection of articular cartilage; (**B**) IF quantification (green scalebar = 200 μm), ns: non-significant, * *p* < 0.05, **** *p* < 0.0001, N = 8.

The SHAM group’s cartilage showed low TXNIP levels (orange-red fluorescence), contrasting with significantly increased TXNIP in the DMM group’s chondrocytes. KD-TXNIP reduced TXNIP expression. OE-TXNIP-AAV injection after DMM surgery notably increased TXNIP levels. Thus, DMM elevates TXNIP in rat chondrocytes, with KD-TXNIP effectively reducing its abnormal expression (Figure 5A,B).

### 3.6. TXNIP Upregulation of Inflammatory Cytokines through the Process of Pyroptosis

**Figure 6:**
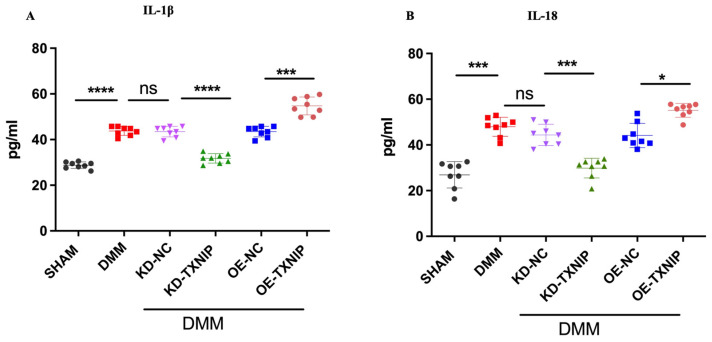
Serum concentration quantification in rats after SHAM/DMM surgery and KD-NC, KD-TXNIP, OE-NC, and OE-TXNIP intra-articular injection of articular cartilage. (**A**) IL-1β; (**B**) IL-18, ns: non-significant, * *p* < 0.05, *** *p* < 0.001, **** *p* < 0.0001, N = 8.

After DMM surgery, the levels of serum inflammatory cytokines IL-1B and IL-18 were elevated and further increased after OE-TXNIP injection. In comparison, the KD-TXNIP intra-articular injection decreased the production of inflammatory factors (Figure 6A,B).

## 4. Discussion

Pyroptosis, a form of programmed cell death associated with inflammation, has been increasingly recognized for its role in various pathological conditions, including OA [5]. Understanding the molecular mechanisms underlying OA progression is crucial for developing targeted therapeutic strategies. TXNIP is a ubiquitously expressed protein that negatively regulates the expression and function of thioredoxin (TXN) [17]. In recent years, TXNIP has garnered significant attention for its diverse roles in energy metabolism and its impact on disease development, particularly in response to various cellular stressors [18]. These newly discovered functions of TXNIP underscore its potential as a therapeutic target. Targeting TXNIP is believed to offer novel therapeutic opportunities and guide future research into its potential for treatment strategies. Previous studies have provided a comprehensive overview of TXNIP’s multiple functions in pathological conditions, highlighting its involvement in diseases such as diabetes, chronic kidney disease, and neurodegenerative disorders [19,20].

By examining the modulation of TXNIP expression and its subsequent effects on pyroptosis in the articular chondrocytes of OA, this study observed that TXNIP expression levels were significantly elevated in the articular chondrocytes of DMM rats compared to the SHAM group. This finding indicates a correlation between TXNIP upregulation and OA progression. Furthermore, the experimental results showed that the destruction of articular cartilage and subchondral bone was reduced in KD-TXNIP rats compared to the OE-TXNIP group.

Through literature studies, TXNIP has been found to play a crucial role in the pathophysiology of various diseases. Scholars have identified associations between TXNIP and several conditions, including cancer [12,21,22,23,24,25,26,27,28,29,30,31,32], atherosclerosis [33], diabetes and its complications [34,35], neurodegenerative and cerebrovascular diseases such as Alzheimer’s disease [36], stroke [37], and subarachnoid hemorrhage [38].

This demonstrates that TXNIP can activate the NLRP3 inflammasome and link it to the pyroptosis pathway [39]. This interaction is particularly significant in the context of inflammatory diseases, diabetes, and other conditions where chronic inflammation plays a critical role [19]. The overexpression of TXNIP can contribute to disease progression. The results showed that intra-articular injection of OE-TXNIP in rats increased the expression levels of IL-18 and IL-1β, whereas intra-articular injection of KD-TXNIP effectively reduced these expression levels in the DMM group of rats. Furthermore, TXNIP expression levels were notably higher in the articular chondrocytes of DMM rats compared to the SHAM group, suggesting a link between TXNIP upregulation and the progression of osteoarthritis (OA). This increase in TXNIP expression was associated with the heightened activation of the pyroptosis pathway, as indicated by elevated levels of pyroptosis-related markers such as NLRP3, caspase-1, and GSDMD, underscoring TXNIP’s pivotal role in mediating pyroptosis in OA.

The intra-articular injection of OE-TXNIP-AAV in DMM rats significantly increased TXNIP expression in articular chondrocytes, exacerbating cartilage damage and enhancing the activation of the pyroptosis pathway, which in turn led to more severe OA symptoms.

On the other hand, treatment with KD-TXNIP had a protective effect against OA progression. KD-TXNIP effectively reduced TXNIP expression levels, decreased the activation of the pyroptosis pathway, and mitigated cartilage degeneration, highlighting the potential therapeutic benefits of targeting TXNIP in OA management.

Histological assessments and immunofluorescence staining revealed that DMM rats with elevated TXNIP expression exhibited significant cartilage surface defects, disorganized chondrocyte arrangement, and increased green fluorescence, indicating high TXNIP levels. Conversely, KD-TXNIP-treated rats had smoother cartilage surfaces, more organized chondrocyte structures, and reduced fluorescence, demonstrating the effectiveness of TXNIP modulation.

In this study, TXNIP expression affected not only articular cartilage destruction, abnormal bone modeling, and bone mass loss in the DMM model but also the level of mechanical pain.

These innovative results emphasize the crucial role of TXNIP in OA progression through the pyroptosis pathway and highlight TXNIP’s potential as a therapeutic target for mitigating OA. This study lays the groundwork for further exploration of TXNIP inhibitors and gene therapy approaches to combating OA and improving joint health.

## 5. Conclusions

This study demonstrated that DMM surgery leads to significant cartilage destruction, subchondral bone loss, and increased levels of serum inflammatory factors (IL-1β and IL-18) in rats. The intra-articular injection of OE-TXNIP-AAV exacerbated these conditions, indicating that TXNIP overexpression worsens OA progression. In contrast, KD-TXNIP-AAV treatment had a protective effect, slowing joint degeneration, reducing cartilage and bone damage, and decreasing levels of inflammatory and pyroptosis markers (MMP-13, NLRP3, caspase-1, GSDMD). Additionally, it partially restored the mechanical pain threshold without affecting the thermal pain threshold.

## Data Availability

The data presented in this study are available upon request from the corresponding author. The data are not publicly available due to patient confidentiality and data privacy regulations.

## Supplementary Materials

The following supporting information can be downloaded at: https://www.mdpi.com/article/10.3390/metabo14090488/s1, Table S1: Primary antibodies used in the IHC experiment; Table S2: Secondary antibody information; Table S3: Primary antibodies used in the IF experiment.
