# Supplementary material for: Thioredoxin-Interacting Protein’s Role in NLRP3 Activation and Osteoarthritis Pathogenesis by Pyroptosis Pathway: In Vivo Study
Source: Metabolites. 2024 Sep 7;14(9):488. doi: 10.3390/metabo14090488 (PMC11433649; doi:10.3390/metabo14090488)
Supplement: Supplementary file 1 [file metabolites-14-00488-s001.zip › metabolites-3154750-supplementary.pdf]

metabolites

Supplementary Materials

Thioredoxin-Interacting Protein's Role in NLRP3 Activation and Osteoarthritis Pathogenesis by Pyroptosis Pathway: In Vivo Study

Ruba Altahla and Xu Tao *

Department of Rehabilitation, Tongji Hospital, Tongji Medical College, Huazhong University of Science and Technology, Wuhan 430030, China; rubamntahla91@gmail.com
* Correspondence: i202122089@hust.edu.cn; Tel.: +86-13-507-129-858

Table S1. Primary antibodies used in the IHC experiment.

Name	Primary antibody source species	Catalog Number	Dilution Ratio	Application	Source
Caspase-1	Rabbit	ER1905-47	1:200	IHC	San ying company, Wuhan
GSDMD	Rabbit	ER1901-37	1:200	IHC	San ying company, Wuhan
NLRP3	Rabbit	ET1610-93	1:200	IHC	San ying company, Wuhan
MMP 13	Rabbit	A-A14	1:200	IHC	San ying company, Wuhan
Coll II	Rabbit	A-A04	1:800	IHC	San ying company, Wuhan

Table S2. Secondary antibody information.

Manufacturer	Article number	Dilution method	Dilution ratio
Jackson	111-035-003	TBST	1:5000
Three Eagles	SA00001-1	TBST	1:5000

Table S3. Primary antibodies used in the IF experiment.

Antibody name	Serial number	Dilution ratio	Manufacturer
TXNIP	ET1705-72	Rabbit, 1:200	San ying company, Wuhan
